# Comparative analysis and optimization of multi-model artificial intelligence for microscopic detection of fungal structures in superficial fungal infections

**DOI:** 10.3389/fcimb.2026.1892808

**Published:** 2026-10-08

**Authors:** Mei Liu, Wenjing He, Linan Ni, Lulu Li, Yuling Tang, Jingwen Tan, Lianjuan Yang

**Affiliations:** 1 Department of Medical Mycology, Center of Infectious Skin Diseases, Shanghai Skin Disease Hospital, Tongji University School of Medicine, Shanghai, China; 2 College of Environmental Science & Engineering, Tongji University, Shanghai, China

**Keywords:** artificial intelligence, deep learning, fungal infection, multi-model detection, YOLOv11

## Abstract

**Background:**

Superficial fungal infections present a growing threat to global public health. Traditional diagnostic techniques, including microscopy and culture, are time-consuming and subjective, often limiting diagnostic accuracy. Artificial intelligence (AI)-driven diagnostic tools offer a promising adjunct to improve efficiency and standardization of microscopic fungal detection.

**Methods:**

We systematically compared six deep learning models, including the You Only Look Once (YOLO) series, the Convolutional Neural Network (CNN) series and the Detection Transformer (DETR). Three evaluation datasets were constructed. Datasets 1 and 2 (derived from 4000 hyphae and 4500 spore images) were used to assess image-level detection using sensitivity, precision and F1 score. Dataset 1 was manually annotated for the YOLO series and DETR; Dataset 2 (unannotated) served as the CNN series’ benchmark. Dataset 3 (1, 870 specimens from 1, 730 patients) was used to evaluate sample-level performance via confusion matrix analysis for the YOLO series and DETR models, employing medical records as the reference standard. The best-performing model was optimized and deployed into an online auxiliary diagnostic platform for real-time clinical decision support.

**Results:**

YOLOv11 outperformed all other evaluated architectures. Detection was generally better at 400× than at 200× magnification. Notably, the F1 score for hyphae detection by YOLOv11 increased from 90.42% (200×) to 92.06% (400×). All models achieved higher accuracy for spores than hyphae. Patient-level confusion-matrix analysis confirmed YOLOv11 superiority. After optimizing an Intersection over Union (IoU) threshold of 0.25 and the maximum confidence (Cmax) strategy, 200× hyphae F1 rose to 97.84% and the positive predictive value (PPV) to 98.27%. Subgroup analysis showed robust performance across different diseases, with tinea capitis reaching the highest F1 score (98.53%).

**Conclusion:**

The optimized YOLOv11 model demonstrates potential as a rapid and standardized supportive tool for the microscopic detection of fungal structures in superficial fungal infections. Integration into an online auxiliary diagnostic platform demonstrates the feasibility of real-time AI-assisted fungal detection, offering a standardized tool to support clinical decision-making. This work supports AI as a potential complement to clinical and laboratory expertise, pending further validation in prospective and multi-center settings, and highlights its potential to streamline fungal infection diagnostic workflows.

## 1 Introduction

Superficial fungal infections represent one of the most prevalent infectious diseases worldwide, and rapid, accurate laboratory diagnosis is essential for guiding effective treatment and preventing disease transmission (Chen et al., 2018; Denning, 2024). These infections affect the skin and its appendages (hair and nails), encompassing a range of conditions, including tinea capitis, corporis, cruris, pedis and onychomycosis-which present with diverse clinical manifestations. Traditional microscopic examination relies heavily on the experience of laboratory personnel, making it subjective, labor-intensive, and vulnerable to fatigue-related errors (Mulet Bayona et al., 2020; Koo et al., 2021). In recent years, artificial intelligence (AI), particularly deep learning, has demonstrated strong potential in medical image analysis. In dermatology, AI-based applications are expanding, with most current efforts concentrated on skin cancer detection (Biswas et al., 2025). Research on the AI-assisted diagnosis of cutaneous fungal infections is still in the initial exploration stage. Recent systematic reviews have comprehensively surveyed this emerging landscape. Shoorgashti et al. systematically reviewed AI-powered microscopic diagnostic techniques for *Candida albicans* detection, highlighting the diversity of deep learning approaches but noting the lack of standardized multi-model evaluation frameworks (Shoorgashti et al., 2026). Similarly, Albuqami et al. reviewed advancements in AI-based diagnostic tools for fungal infections, emphasizing that most existing studies focus on single-model evaluation with limited dataset sizes and lack systematic comparison across architectures and magnification levels (Albuqami et al., 2026). In addition, Han et al. developed a region-based convolutional neural network (R-CNN) model for diagnosing onychomycosis, which outperformed most dermatologists (Han et al., 2018). Tahir et al. used a CNN to classify five types of fungal spores, achieving a high accuracy rate of 94.8% (Tahir et al., 2018). In China, Huang Huaiqiu’s team developed an automated microscope system based on deep learning that demonstrated high sensitivity (97.2%) and specificity (99.4%) in detecting filamentous fungi and yeasts (Gao et al., 2021). Recent systematic reviews have highlighted the growing role of AI in fungal diagnostics, yet they also emphasize the need for comprehensive multi-model comparisons and standardized evaluation frameworks. Therefore, this study aims to address this gap by systematically comparing six state-of-the-art deep learning architectures at 200x and 400x magnifications, optimizing the best-performing model, and validating its clinical utility through patient-level analysis and deployment on an online auxiliary diagnostic platform.

## 2 Materials and methods

All image data were collected at Shanghai Skin Disease Hospital between April 1, 2025 and November 30, 2025. The study protocol was approved by the hospital’s Ethics Committee (2026-04) and conducted in accordance with the Declaration of Helsinki. The overall workflow of this study is illustrated in **Figure 1**.

**Figure 1:**
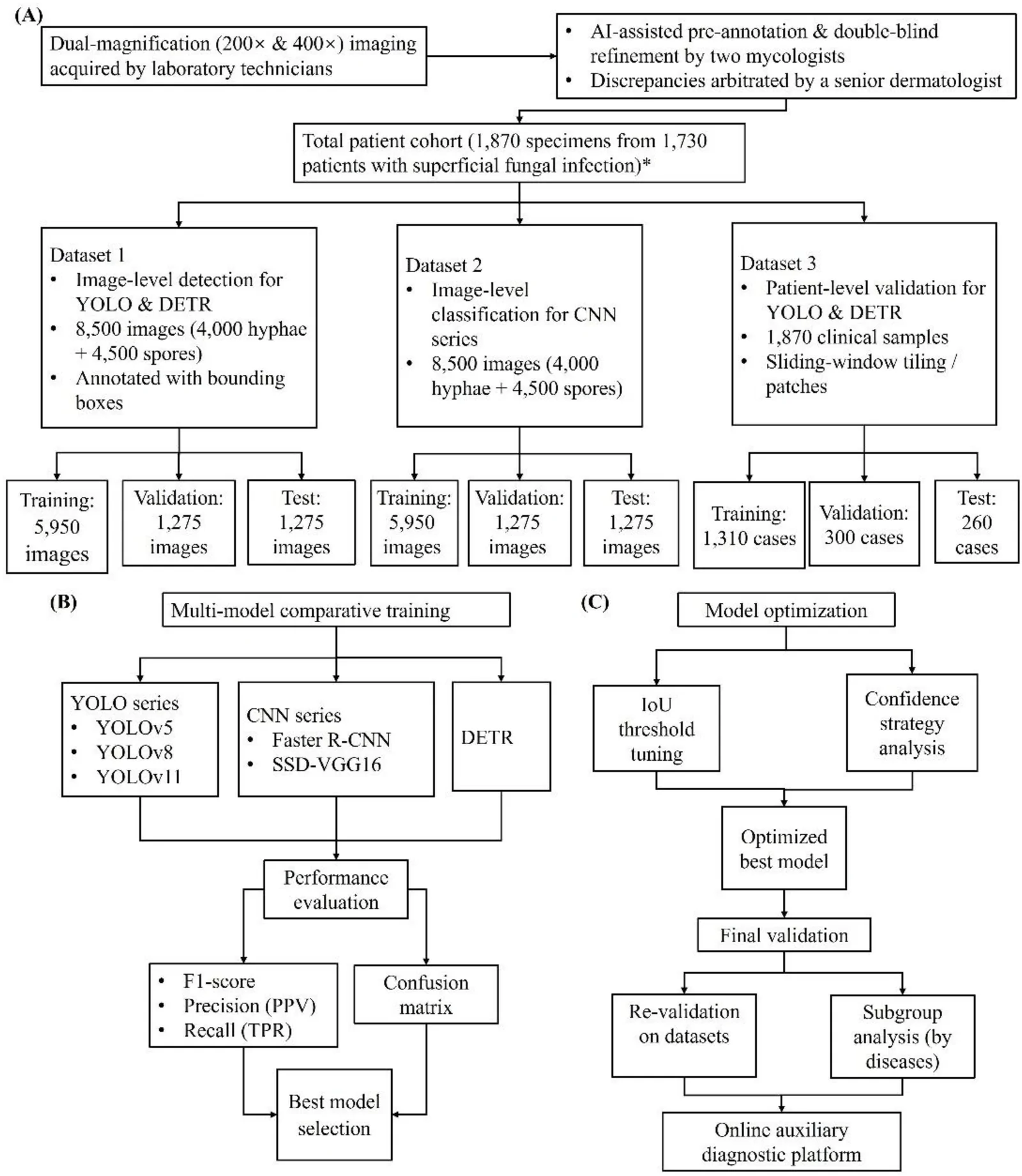
The overall workflow of the AI-based fungal detection study: **(A)** data collection and specimen preparation, **(B)** multi-model comparative training and statistical selection of the optimal baseline, **(C)** targeted model optimization and deployment on an online auxiliary diagnostic platform. *Datasets 1, 2, and 3 were derived from a single cohort of 1, 870 specimens from 1, 730 patients for three distinct tasks: Dataset 1 (8, 500 annotated images) for object detection, Dataset 2 (8, 500 unannotated images) for CNN classification, and Dataset 3 (1, 870 samples) for patient-level clinical validation. YOLO, You Only Look Once; CNN, Convolutional Neural Network; DETR, Detection Transformer; Faster R-CNN, Faster Region-based Convolutional Neural Network; SSD-VGG16, Single Shot MultiBox Detector with VGG16 backbone; TPR, True Positive Rate; PPV, Positive Predictive Value; IoU, Intersection over Union.

### 2.1 Model overview

This study employed two approaches to determine image class: image classification (CNN-based models) and object detection (YOLO series and DETR). Image classification evaluates the entire micrograph and returns a binary (positive or negative) result, offering a simpler and more direct process than object detection. In contrast, object detection is more sophisticated. It localizes specific targets using bounding boxes to define their size and position; a discriminator then determines the sample’s status based on the presence and probability of these detected objects. The architectures of the six evaluated models are shown in **Figure 2**.

**Figure 2:**
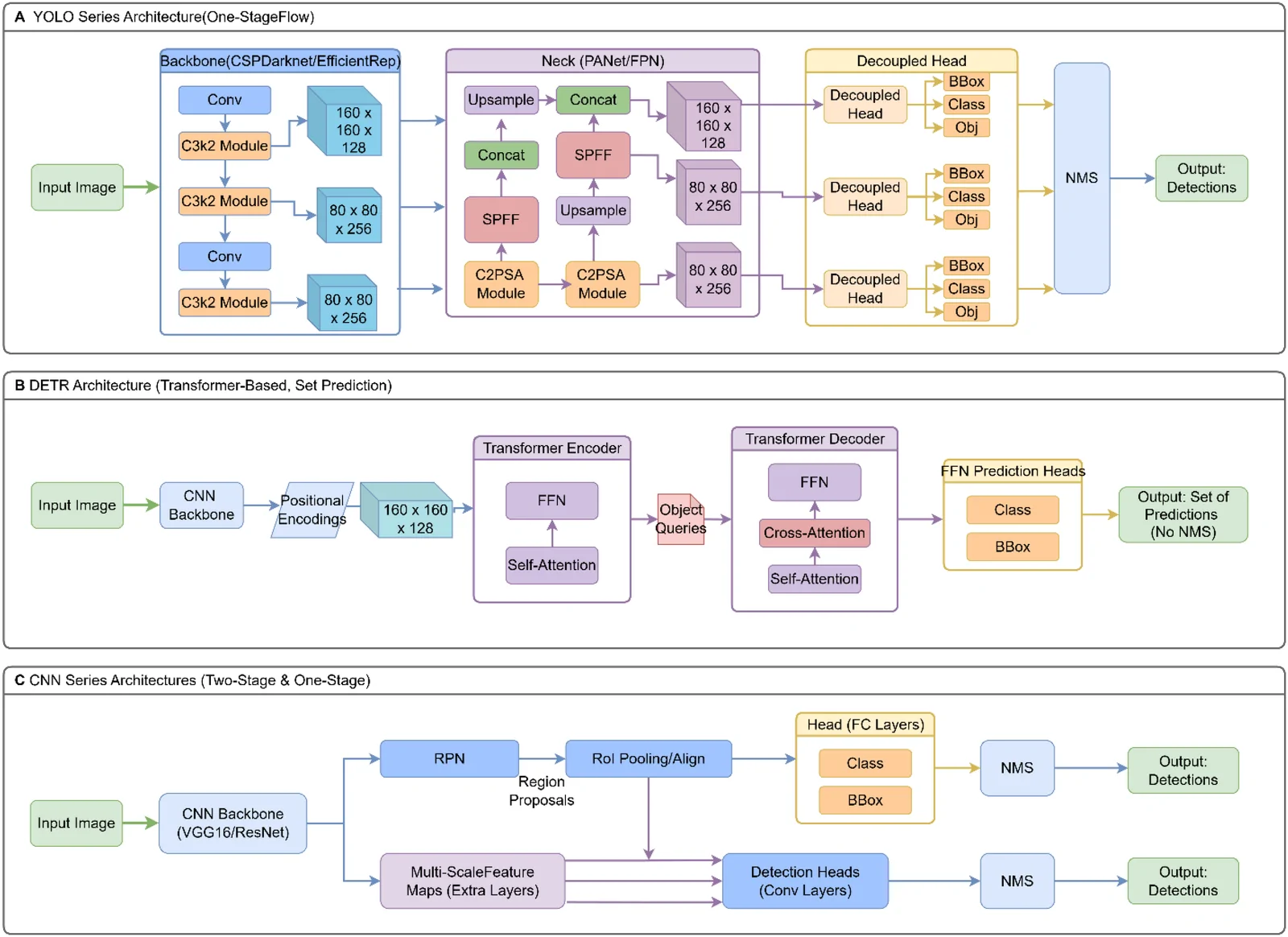
Architectures of the deep learning models evaluated in this study: **(A)** YOLO series, **(B)** DETR, **(C)** the CNN series. CSPDarknet, Cross Stage Partial Darknet; EfficientRep, Efficient Reparameterized Backbone; Conv, Convolutional Layer; C3k2, Cross Stage Partial Bottleneck with 3 convolutions version 2; PANet, Path Aggregation Network; FPN, Feature Pyramid Network; Concat, Concatenation; SPFF, Spatial Pyramid Pooling Fast; C2PSA, Contextual Post-Self-Attention; BBox, Bounding Box; Obj, Objectness Score; NMS, Non-Maximum Suppression; FFN, Feed-Forward Network; RPN, Region Proposal Network.

We assessed two established CNN-based models: SSD-VGG16 and Faster R-CNN. The SSD-VGG16 model integrates the Single Shot MultiBox Detector (SSD) framework with a Visual Geometry Group 16 (VGG16) backbone to achieve balanced detection performance (Simonyan and Zisserman, 2014). Faster R-CNN was designed to overcome the limitations of R-CNN arising from its high computational complexity (Girshick, 2015). The YOLO series represents state-of-the-art one-stage detectors, characterized by high inference speeds and an optimal balance between accuracy and efficiency, making them well-suited for real-time clinical applications (Redmon et al., 2015). Finally, we incorporated DETR, a transformer-based model that utilizes self-attention mechanisms to capture global context information, providing specific advantages when processing images with complex background noise (Carion et al., 2020).

### 2.2 Images collection

Clinical specimens were collected from the margins of active lesions using a sterile scalpel and transferred onto microscope slides. A drop of fluorescent stain solution (Chongqing Tianhai Medical Equipment Co., Ltd., Chongqing, China) was added, followed by placement of a coverslip with gentle pressure to ensure even distribution. Dual-magnification microscopic imaging was systematically collected by trained laboratory technicians with at least 5 years of experience in fungal microscopy at Shanghai Skin Disease Hospital. A standardized acquisition protocol was followed: initial screening at 200× to identify suspected fungal structures, followed by detailed morphological examination at 400×. Images were captured using a microscope (Olympus BX53, Tokyo, Japan) equipped with a microscope camera (MShot MDX10, Guangzhou, China) and microscope software (MShot Image Analysis System V1.1.4A, Guangzhou, China). Corresponding patient information and diagnostic results were recorded for each specimen. All acquired images were first screened by two senior mycologists (each with >5 years of clinical diagnostic experience) to exclude unqualified micrographs that were out of focus, under- or overexposed, or poorly centered. To optimize annotation precision and efficiency, eligible images then underwent a collaborative multi-tier workflow: fungal structures were initially pre-annotated by AI, followed by rigorous, double-blind manual verification and boundary refinement by the two mycologists. Any ambiguous annotations or inter-observer discrepancies were adjudicated by an independent senior dermatologist to ensure inter-rater consistency. Inter-observer agreement between the two primary annotators—evaluated on an independently double-annotated subset of 4, 000 images—was substantial (Cohen’s κ = 0.87). The few discrepant cases (n=43, accounting for 1.1% of the 4, 000 reviewed images) were subsequently adjudicated by a third senior expert to establish the definitive consensus ground truth.

### 2.3 Dataset construction

To evaluate the deep learning models from both technical and clinical perspectives, three task-specific datasets were established from a unified master repository of 1, 870 specimens from1, 730 patients: (1) Dataset 1 (8, 500 annotated patches) for object detection frameworks; (2) Dataset 2 (8, 500 unannotated patches) for CNN-based classification baselines; and (3) Dataset 3 (1, 870 specimens from 1, 730 patients) for patient-level clinical concordance validation. Using full-image inputs often involves an excessively large receptive field, which can hinder the capture of local, fine-grained details and lead to suboptimal data utilization. To address this, each original microscopic image was divided into uniform patches, and valid regions were manually selected for inclusion. Dataset 1 was used for training and validating the YOLO series and DETR frameworks, which require precise target localization via bounding boxes. Since these object detection models share a common requirement for target localization via bounding boxes, a single set of expert-verified annotations was applied universally across these frameworks. In contrast, tailored for CNN-based architectures and requiring no bounding box annotations, Dataset 2 utilized the same 8, 500 images to align with the unique technical baselines of CNN models. The primary objective of these image-level detections was to establish a robust technical benchmark. By quantifying each model’s capacity to precisely localize and classify individual fungal elements-which often exhibit significant morphological heterogeneity and irregular structures-these datasets provided a fundamental measure of the algorithms’ underlying technical stability.

While image-level metrics are crucial for algorithmic refinement, clinical practice fundamentally prioritizes patient-level diagnosis over the analysis of isolated fields of view. Therefore, Dataset 3 was constructed as an independent clinical validation cohort, comprising 1, 870 specimens from 1, 730 patients. Ground truth was defined using a composite reference standard established by board-certified dermatologists, requiring concordant multi-modal evidence: (1) macroscopic clinical presentation (characteristic lesion morphology and distribution patterns), (2) patient history and symptoms, (3) direct fluorescent microscopic confirmation of fungal structures (hyphae and/or spores) verified through double-blind review and senior arbitration (L.J.Y.), and (4) response to antifungal therapy. Specimens negative by both clinical presentation and direct microscopy served as negative controls. As general-purpose object detection frameworks originally designed for natural images, SSD-VGG16 and Faster R-CNN showed unsatisfactory performance in detecting small, low-contrast lesions in medical images, accompanied by high rates of missed and false detections, low inference efficiency and complex post-processing steps. These limitations failed to meet the stringent requirements for clinical deployment in terms of safety, reliability and operational efficiency. This patient-level dataset enables a direct and comprehensive assessment of the diagnostic efficacy of the DETR and YOLO series models against the gold-standard clinical outcome. All datasets were partitioned into training, validation, and test sets at a 7:1.5:1.5 ratio, ensuring that all images and patches derived from a single specimen were allocated exclusively to one subset; moreover, all specimens obtained from the same patient were assigned to the same subset, precluding any patient-level overlap between training, validation, and test partitions. Specifically, Dataset 3 was partitioned into a training set of 1, 310 specimens (14, 000 images), a validation set of 300 specimens (3, 000 images), and a test set of 260 specimens (3, 000 images), yielding a total of 20, 000 microscopic images, with an average of roughly 10 field images per specimen. This partitioning precludes any possibility of data leakage across subsets.

### 2.4 Model training

The model development pipeline was structured into four integrated modules: data preprocessing and augmentation, feature extraction (backbone), feature fusion (neck) and prediction (head).

The process began with the establishment of a robust technical benchmark. The CNN-based architectures were trained on Dataset 2, which consisted of localized sub-images generated via sliding-window tiling of clinical images, to establish a stable performance baseline. To enhance model robustness against the irregular morphologies of fungal elements, advanced data augmentation techniques, including Mosaic and MixUp, were applied across all datasets. All candidate models were trained at a consistent input resolution of 640 × 640 pixels for 200 to 300 epochs. The subsequent feature extraction stage employed optimized backbones to capture hierarchical fungal features. The YOLO series utilized Cross Stage Partial Darknet (CSPDarknet) or Efficient Reparameterized (EfficientRep) architectures, while the CNN-based models used standard VGG16 or Residual Network (ResNet) frameworks, the latter being foundational architectures previously validated for fungal detection tasks. Feature fusion was then performed to integrate multi-scale information. The YOLO series integrated Path Aggregation Networks (PANet) or Feature Pyramid Networks (FPN), whereas the DETR model utilized Transformer-based self-attention mechanisms to capture global contextual dependencies. The Faster R-CNN architecture employed an RPN for generating candidate regions. The final phase involved prediction and optimization. Models were refined using Complete Intersection over Union (CIoU) loss for bounding box regression and binary cross-entropy for classification. Optimization strategies were tailored to architectural requirements. YOLOv8, YOLOv11 and DETR used the Adam with Weight Decay (AdamW) algorithm, while YOLOv5 and the CNN series used Stochastic Gradient Descent (SGD) with a momentum of 0.937. All training pipelines included a warm-up phase of 2–4 epochs and maintained weight decay between 0.0006 and 0.0008.

### 2.5 Performance evaluation

The six models the three datasets with distinct objectives. For Datasets 1 and 2, designed for localized detection, model performance was assessed using True Positive Rate/Recall (TPR), Positive Predictive Value/Precision (PPV), and the F1 score calculated as follows (Equations 1–3):

(1) $$TPR(\%)=\frac{TP}{TP+FN}*100$$

(2) $$PPV(\%)=\frac{TP}{TP+FP}*100$$

(3) $$F1\;score(\%)=2\frac{PPV*TPR}{PPV+TPR}*100$$

Here, True Positives (TP) and True Negatives (TN) represent correct predictions, False Positives (FP) and False Negatives (FN) represent errors. While an ideal diagnostic test would exhibit 100% sensitivity and specificity (Umemneku Chikere et al., 2019), practical methods often involve a trade-off, which is quantified by the F1 score. For each experimental condition (specific target structure and magnification level), the model achieving the maximum F1-score was determined as the top-performing architecture, as indicated in the comparative tables to facilitate direct performance benchmarking.

Dataset 3, the patient-level set, was used to assess clinical utility. The model’s automated sample-level diagnosis (positive/negative) was compared against the established composite clinical reference standard using a confusion matrix, providing a direct measure of real-world diagnostic accuracy.

### 2.6 Statistical analysis

Inter-observer agreement between the two annotators was quantified using Cohen’s kappa coefficient (κ = 0.87, indicating substantial agreement). For patient-level evaluation (Dataset 3), the diagnostic performance of YOLOv11 was compared with that of the benchmark models (YOLOv8, YOLOv5, and DETR) using McNemar’s test for paired proportions and DeLong’s test for comparing ROC curves.

### 2.7 Model optimization

Following initial evaluation, the best-performing model underwent parameter optimization focusing on two key clinical translation aspects.

First, IoU threshold optimization was performed. To determine the optimal criterion for matching a detected bounding box to a ground-truth annotation, the model’s PPV, TPR and F1 score were evaluated across IoU thresholds from 0.25 to 0.75 (in increments of 0.25). PR curves were generated to visualize the performance trade-off across confidence thresholds.

Second, image-level confidence aggregation was analyzed. Since the final clinical output is an image-or sample-level classification, three strategies for aggregating confidence scores from all detected bounding boxes within an image were evaluated: Cmax (maximum confidence), Cmin (minimum confidence) and Cavg (average confidence). These were derived from the detection box confidence scores within each image. The minimum, maximum and average probabilities were calculated as follows (Equations 4–6):

(4) $$C_{max}=\underset{\text{k }=\;1,\ldots ,\text{n}}{\max}C_{k}$$

(5) $$C_{min}=\underset{\text{k }=\;1,\ldots ,\text{n}}{\min}C_{k}$$

(6) $$C_{avg}=\frac{1}{n}\sum_{k=1}^{n}C_{k}$$

Receiver Operating Characteristic (ROC) analysis was conducted using the MATLAB perfcurve function to identify the optimal diagnostic strategy and classification threshold.

### 2.8 Validation and platform construction

For validation, the optimized model was then re-evaluated to quantify its performance improvement over the baseline model. Subgroup analyses were further performed according to disease categories to evaluate the model’s diagnostic efficacy for specific conditions. Finally, an online auxiliary diagnostic platform was implemented based on the core algorithm, enabling clinicians to upload images and obtain AI-generated diagnostic reports in real time.

## 3 Results

### 3.1 Demographic characteristics and disease distribution

The cohort consisted of 1092 males (63.12%) and 638 females (36.88%), with an age range of 6 to 97 years. The median age was 39 years (interquartile range: 28-59), and the distribution of age deviated significantly from normality (P < 0.05).

Specimens represented a range of common superficial fungal infections. The most prevalent diagnoses were tinea cruris (n=453), tinea corporis (n=411), and onychomycosis (n=348), with pityriasis versicolor (n=272) and tinea pedis (n=226) ranking next. Less common conditions included tinea faciei (n=66), tinea manuum (n=50), genital candidiasis (n=34) and tinea capitis (n=10).

### 3.2 Performance comparison of different models

The baseline performance of the six deep learning architectures was systematically evaluated on Datasets 1 and 2 at 200× and 400× magnifications (**Table 1**; **Figure 3**).

**Table 1:** Comparative baseline performance of six models for hyphae and spore detection.

Model	Target structure	Magnification	PPV (%)	TPR (%)	F1 (%)
YOLOv5	Hyphae	200	88.38	87.90	88.14
		400	93.18	88.73	90.90
	Spore	200	91.19	93.42	92.29
		400	95.46	96.08	95.77
YOLOv8	Hyphae	200	89.77	89.24	89.50
		400	92.09	90.26	91.17
	Spore	200	93.15	93.06	93.10
		400	97.38	96.16	96.77
YOLOv11	Hyphae	200	90.88	89.97	90.42
		400	93.38	90.78	92.06
	Spore	200	95.61	93.70	94.65
		400	97.13	96.35	96.74
DETR	Hyphae	200	86.81	82.17	84.43
		400	87.39	83.22	85.25
	Spore	200	93.97	93.41	93.69
		400	97.45	94.34	95.87
Faster R-CNN	Hyphae	200	62.48	98.81	76.55
		400	75.87	97.40	85.30
	Spore	200	80.91	99.26	89.15
		400	92.79	98.91	95.75
SSD-VGG16	Hyphae	200	86.57	58.77	70.01
		400	85.26	77.83	81.38
	Spore	200	94.01	62.59	75.15
		400	94.30	90.05	92.13

*Values in red text indicate the highest F1-score achieved under each experimental condition (specific target and magnification level), representing the top-performing model architecture.

**Figure 3:**
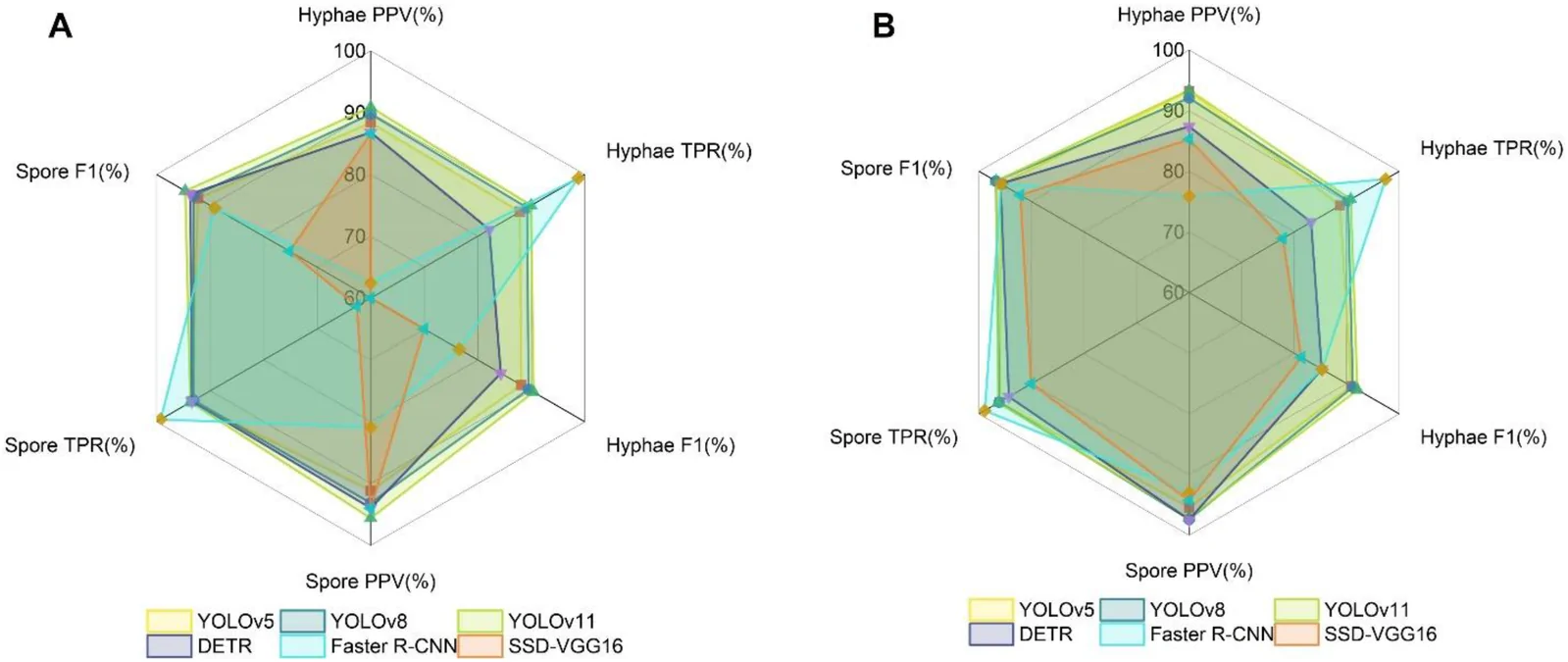
Radar charts illustrating the detection performance for hyphae and spores: **(A)** Radar Chart for 200 × magnification, **(B)** Radar Chart for 400 × magnification. F1, F1 score.

With respect to magnification effects, most models achieved better performance at 400× than at 200×. For instance, in YOLOv8, PPV and TPR for spore detection increased from 93.15% and 93.06% at 200× to 97.38% and 96.16% at 400×, respectively. Similarly, the TPR of spore detection using YOLOv11 rose from 93.70% at 200× to 96.35% at 400×. Notably, Faster Region-based Convolutional Neural Network (Faster R-CNN) was the model to exhibit decreased performance at 400×, with the hyphal TPR declining from 98.81% to 97.40% and spore TPR from 99.26% to 98.91%.

Further analysis of detection targets showed that, at identical magnification, all models performed significantly better in spore detection than in hyphal detection. Taking the DETR model as an example, its PPV for spore detection at 200× reached 93.97%, substantially exceeding that for hyphae (86.81%).

In terms of overall diagnostic performance, CNN-based models, including Faster R-CNN and Single Shot MultiBox Detector with Visual Geometry Group 16 backbone (SSD-VGG16), generally underperformed compared with the YOLO series. Although Faster R-CNN achieved high TPR for both hyphae and spores, this advantage came at the expense of precision (**Figure 3**). Specifically, at 200×, its hyphal detection yielded a TPR of 98.81% but a PPV of only 62.48%, reflecting an overly sensitive detection strategy prone to false positives. The performance of SSD-VGG16 was severely limited by low sensitivity, with its extremely low TPR values acting as a bottleneck for the overall F1 scores. For example, its hyphal TPR at 200× was 58.77%, corresponding to an F1 score of merely 70.01%. In contrast, the DETR model exhibited competitive performance in spore detection at 400×, with a PPV of 97.45% marginally surpassing that of YOLOv11 (97.13%). Nevertheless, its overall diagnostic efficacy across all other metrics remained inferior to the YOLO series.

The YOLO series demonstrated clear advantages in overall diagnostic efficacy, with distinct performance variations across versions. YOLOv5 was the weakest performer within the series, with F1 scores for hyphae and spores at 200× of 88.14% and 92.29%, respectively-the lowest among all YOLO variants. In contrast, YOLOv8 and YOLOv11 showed strong and competitive performance. YOLOv11 generally outperformed YOLOv8 across most core metrics, with the only exception being spore detection at 400×, where YOLOv8 achieved a slightly higher PPV (97.38%) than YOLOv11 (97.13%). At 200×, however, YOLOv11 attained a higher spore PPV (95.61%) than YOLOv8 (93.15%).

Finally, a radar chart summarized the comprehensive performance of the six models across six evaluation dimensions (**Figure 3**). The YOLO series exhibited distinct superiority in overall performance, as reflected by its markedly larger enclosed area. While YOLOv5 remained at a relatively low baseline level, YOLOv11 achieved the most balanced and superior overall performance. In comparison, Faster R-CNN exhibited an imbalanced profile characterized by high TPR but low PPV, and SSD-VGG16 showed the lowest overall detection efficacy. Although DETR was highly competitive in the 400× spore recognition, its overall diagnostic performance was still inferior to the YOLO series. At the image level (Datasets 1 and 2), YOLOv11 demonstrated superior detection performance across most experimental conditions (different target structures and magnification levels), achieving peak F1-scores as highlighted in **Table 1**.

### 3.3 Patient-level diagnostic validation and model selection

To assess the clinical utility of the models, a diagnostic analysis was conducted at patient level using Dataset 3 (**Figure 4**). Both YOLOv11 and YOLOv8 achieved an identical number of true positives (TP, 213), indicating equivalent diagnostic sensitivity. However, YOLOv11 generated only 3 false positives (FP), whereas YOLOv8 produced 10. This suggests that YOLOv8 exhibited a higher tendency to misclassify artifacts as fungal structures, which could lead to clinical over-diagnosis. Consequently, YOLOv11 achieved a significantly higher specificity of 93.18% and an overall accuracy of 97.69%, markedly outperforming YOLOv8 (specificity: 77.27%; accuracy: 95.00%). YOLOv5 and DETR demonstrated limited clinical applicability due to excessively high false-positive rates (70.45% and 100.00%, respectively) and low true-negative rates (29.55% and 0.00%, respectively). Pairwise McNemar’s tests were performed to assess statistical differences in diagnostic performance among the models (**Table 2**). Consistent with the baseline metrics reported in **Table 1**, YOLOv11 achieved significantly higher diagnostic accuracy than both YOLOv5 (P<0.001) and DETR (P<0.001). In contrast, the performance difference between YOLOv11 and YOLOv8 did not reach statistical significance (P = 0.118), indicating comparable diagnostic accuracy between the two latest-generation architectures. However, YOLOv11 was the only model with perfectly balanced false positives and false negatives (3/3). Furthermore, DeLong’s test showed that the patient-level AUC of YOLOv11 (0.959) was significantly higher than that of YOLOv8 (0.879; P = 0.015), indicating superior overall discriminative ability (**Table 2**, **Table 3**).

**Figure 4:**
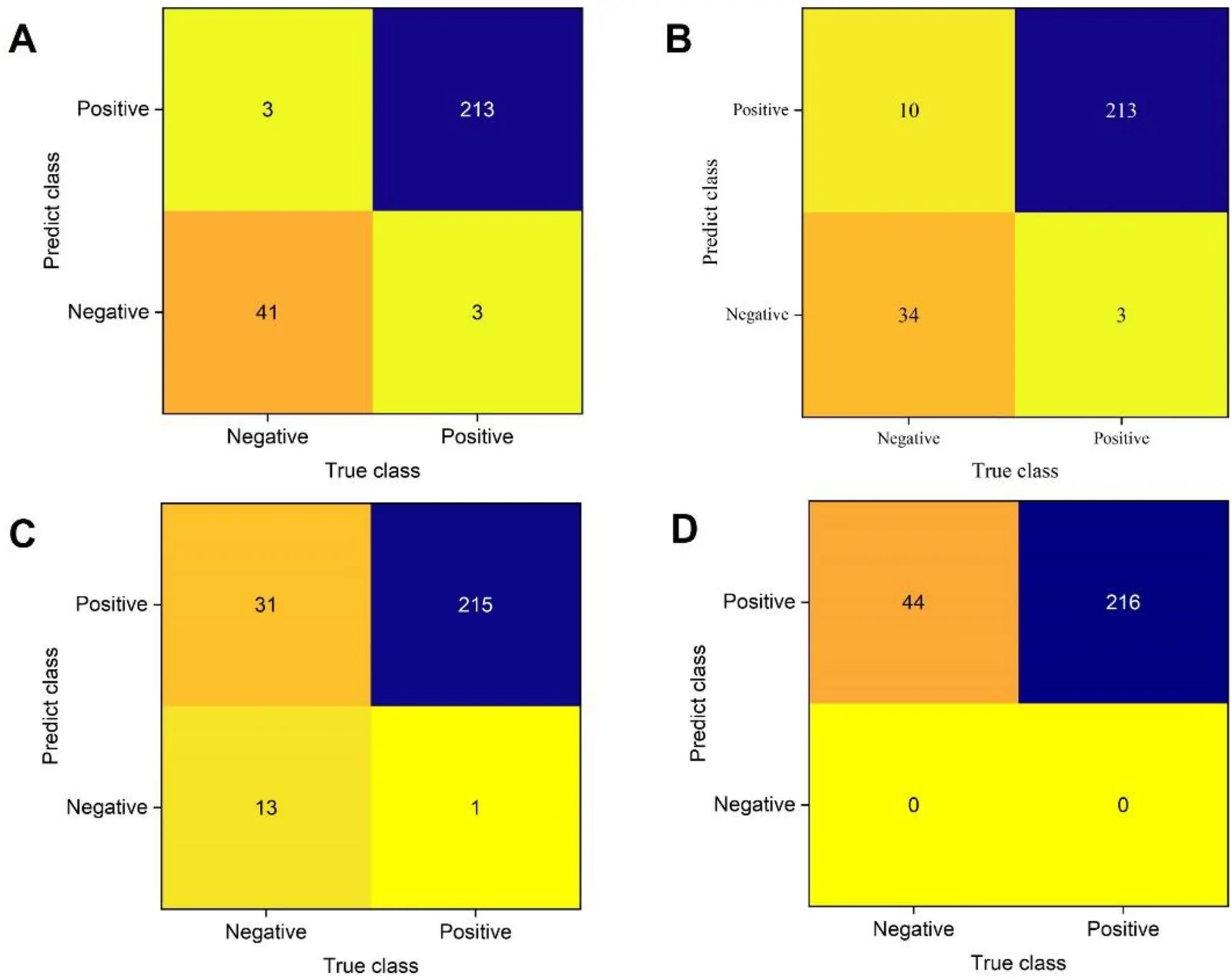
Patient-level diagnostic performance visualized through confusion matrices: **(A)**YOLOv11, **(B)** YOLOv8, **(C)** YOLOv5, **(D)** DETR.

**Table 2:** Pairwise comparisons of patient-level diagnostic performance among deep learning models using McNemar’s test and DeLong’s test.

Model comparison	McNemar test P value	DeLong’s test P value
YOLOv5 & YOLOv11	<0.001	<0.001
YOLOv5 & YOLOv8	<0.001	<0.001
YOLOv8 & YOLOv11	0.118	0.015
YOLOv5 & DETR	<0.001	<0.001
YOLOv8 & DETR	<0.001	<0.001
YOLOv11 & DETR	<0.001	<0.001

**Table 3:** Patient-level discriminative performance of deep learning models evaluated using DeLong’s method on Dataset 3.

Model	AUC	95% CI
DETR	0.500	-*
YOLOv5	0.645	0.577-0.714
YOLOv8	0.879	0.816-0.943
YOLOv11	0.959	0.921-0.997

*The 95% CI for DETR could not be computed because the model yielded constant predictions across all test cases (all specimens were classified as positive).

Based on the comprehensive assessment on Datasets 1 and 2, as well as superior specificity in the patient-level validation of Dataset 3, YOLOv11 was selected as the optimal model for subsequent analyses.

### 3.4 Model optimization analysis

To further enhance the performance of YOLOv11, the effects of IoU thresholds and image-level confidence strategies were investigated (**Table 4**, **Figure 5**). First, evaluation of IoU thresholds (0.25, 0.5, 0.75) revealed that peak performance was achieved at IoU=0.25. At this threshold, hyphae detection reached a TPR of 90.16%, a PPV of 87.62%, and an F1 score of 88.87%, while spore detection achieved a TPR of 97.28% and a PPV of 93.91%. Increasing the IoU threshold from 0.25 to 0.75 resulted in consistent performance degradation: the hyphae F1 score dropped from 88.87% to 80.33%, and the spores F1 score decreased from 95.56% to 91.47%. Precision-recall (PR) curves further supported these results, showing that the area under the curve was maximized at an IoU of 0.25 (**Figures 5A, B**). Secondly, ROC analysis of confidence aggregation strategies demonstrated that Cmax strategy provided the strongest discriminative power (**Figures 5C, D**). Under the optimal IoU threshold, the area under the curve (AUC) for hyphae detection using Cmax was 0.9910, significantly higher than those of Cmin (0.7875) and Cavg (0.9779). For spore detection, Cmax achieved an AUC of 0.9950.

**Table 4:** Impact of different IoU thresholds on PPV, TPR, and F1 score for hyphae and spores.

Target structure	IoU	PPV (%)	TPR (%)	F1 (%)
Hyphae	0.25	87.62	90.16	88.87
	0.50	86.91	89.43	88.16
	0.75	79.20	81.50	80.33
Spore	0.25	93.91	97.28	95.56
	0.50	93.62	96.97	95.27
	0.75	89.89	93.11	91.47

**Figure 5:**
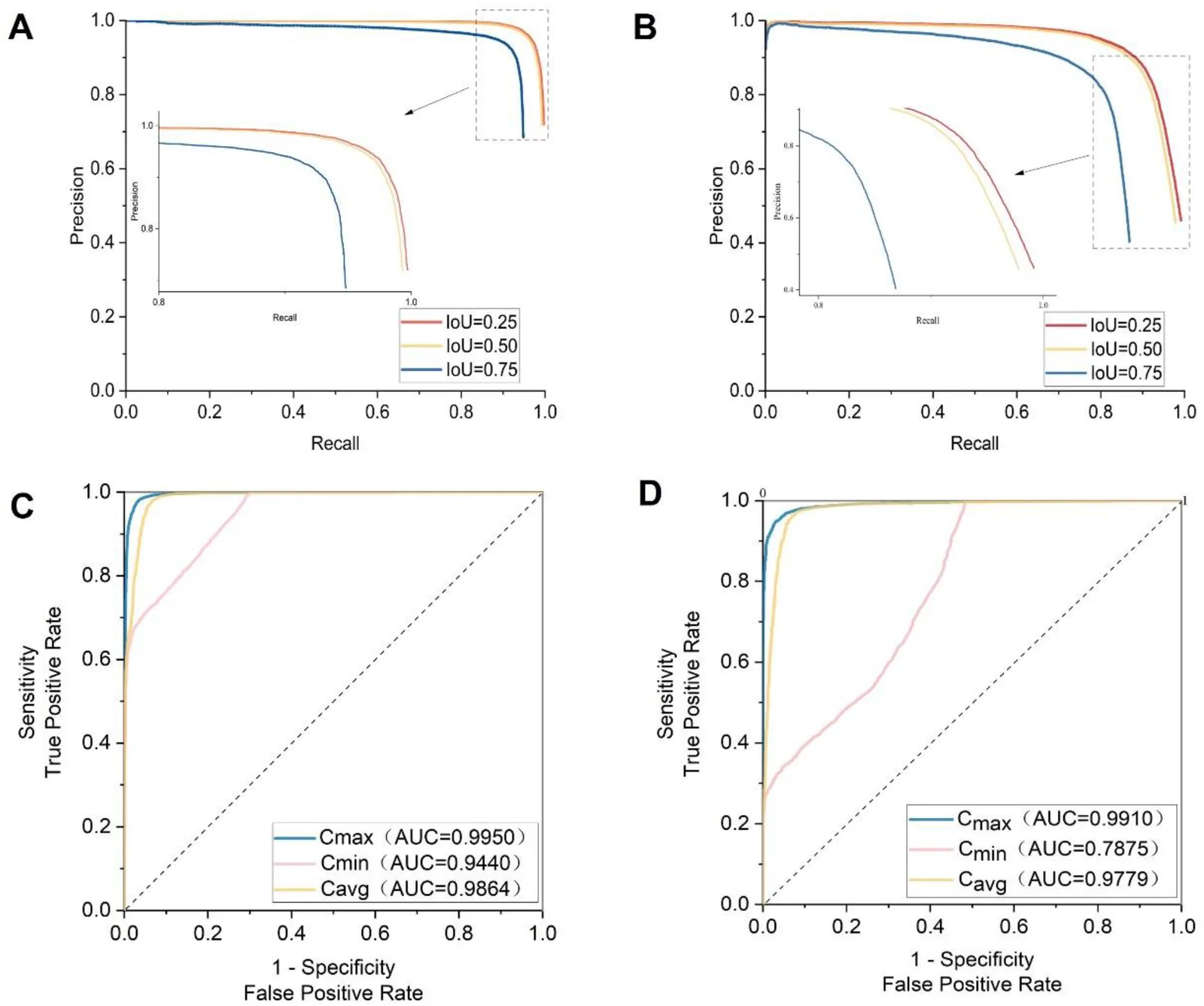
Precision-recall (PR) curves comparing different IoU thresholds **(A, B)** and receiver operating characteristic (ROC) curves with different probabilities (IoU=0.25) **(C, D)**: **(A)** PR curves for spore detection, **(B)** PR curves for hyphae detection, **(C)** ROC curves for spore detection, **(D)** ROC curves for hyphae detection.

### 3.5 Performance of the optimized model and subgroup analysis by diseases

Application of the optimized parameters (IoU=0.25, Cmax strategy) to YOLOv11 resulted in significant performance improvements (**Table 5**), with the magnitude of changes visualized in the heatmap (**Figure 6**). Overall, the optimized model exhibited improvements across nearly all detection metrics. Notably, performance at 200× magnification was remarkably improved: the hyphae F1 score increased by 7.42% to 97.84%, driven by a 7.44% rise in TPR and a 7.39% increase in PPV. The spore PPV at 200× also improved from 95.61% to 97.29%. The only exceptions were marginal decreases of 0.46% in TPR and 0.23% in F1 score for spore detection at 400×.

**Table 5:** PPV, TPR, and F1 score of hyphae and spores based on optimized YOLOv11.

Target structure	Magnification	PPV (%)	TPR (%)	F1 (%)
Hyphae	200	98.27	97.41	97.84
	400	97.66	96.77	97.21
Spore	200	97.29	98.11	97.70
	400	97.14	95.89	96.51

**Figure 6:**
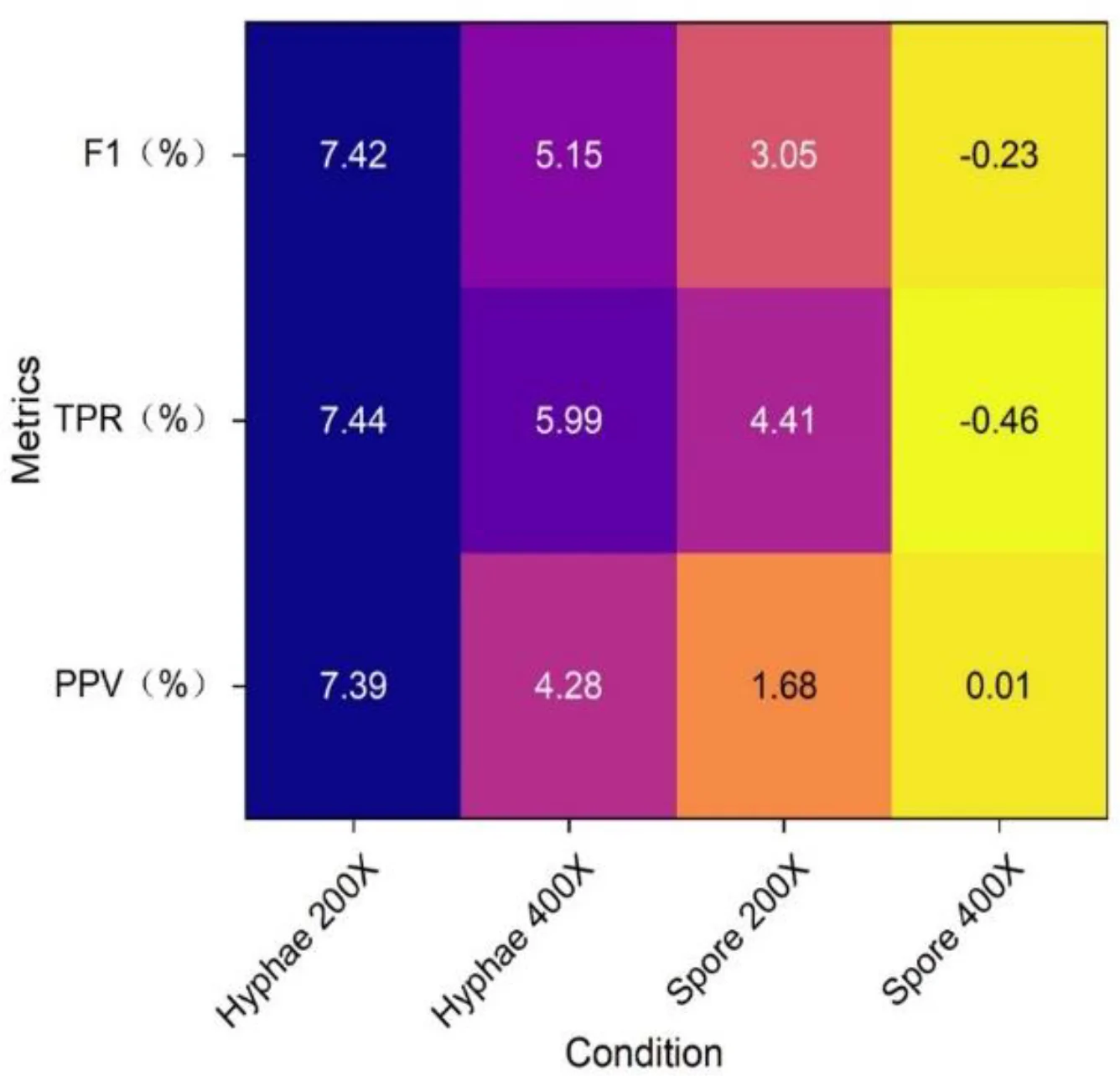
Heatmap visualization of performance changes following model optimization. Visualization of percentage increases in F1 score, TPR and PPV across different detection conditions.

Subgroup analysis revealed robust diagnostic performance across different diseases (**Figure 7**). The model performed best for tinea capitis, with a PPV of 97.10%, TPR of 100.00% and F1 score of 98.53%. High performance was also observed for tinea pedis and genital candidiasis, with F1 scores of 94.95% and 94.05%, respectively. Overall, F1 scores consistently exceeded 90% for most fungal infection subtypes, demonstrating high reliability. The only exception was tinea manuum, which exhibited the lowest performance (F1 = 80.25%).

**Figure 7:**
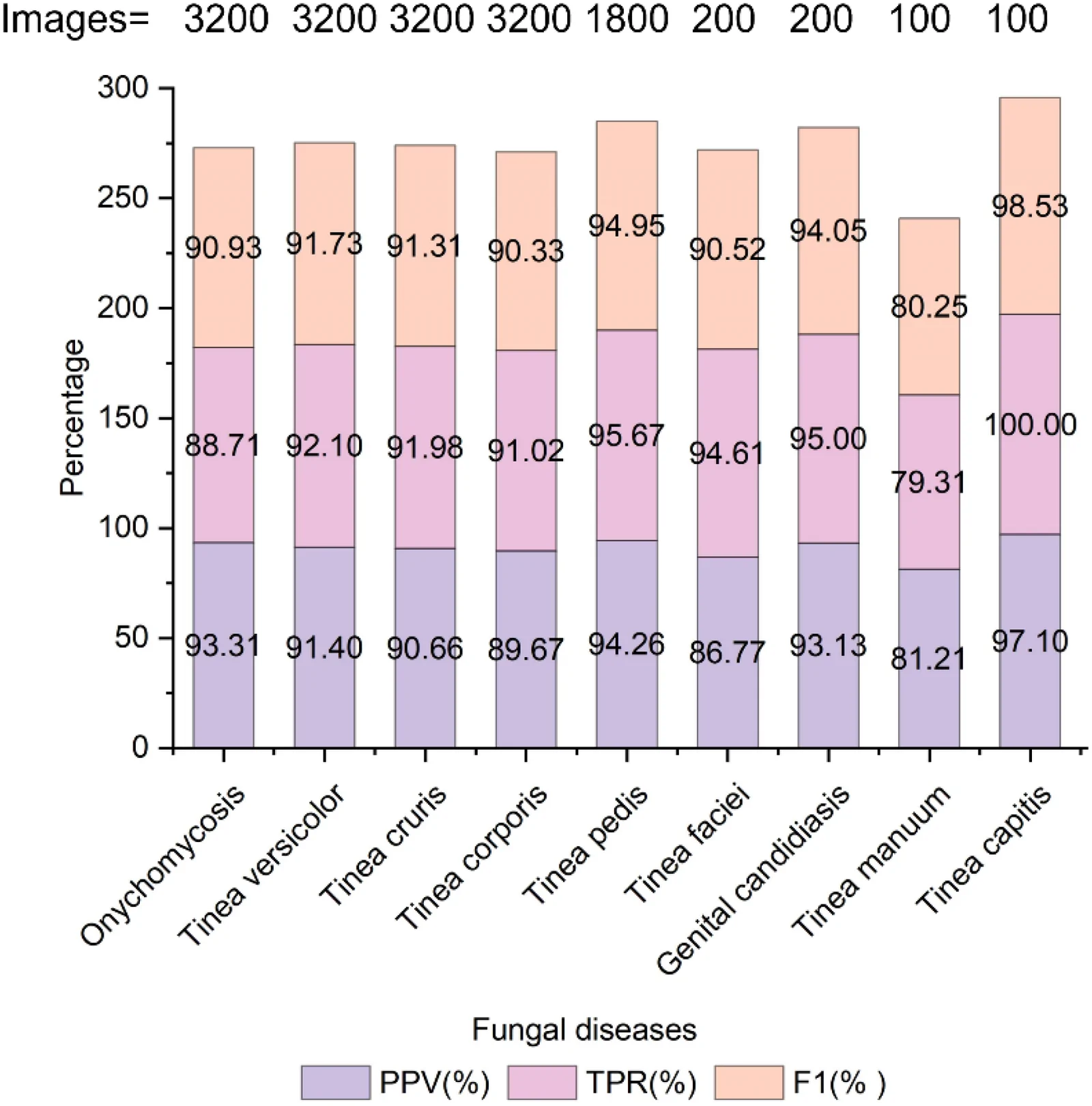
Comparison of YOLOv11 model’s detection performance for different skin fungal diseases.

### 3.6 Establishment of an online auxiliary diagnostic platform

Leveraging the optimized YOLOv11 algorithm, we developed and deployed an online fungal detection platform (**Figure 8**). The platform is designed for high-throughput clinical scenarios, enabling the upload of microscopic images for immediate automated analysis. It generates comprehensive diagnostic reports that comprise a binary positive/negative classification result, enhanced visualization with bounding boxes that localize fungal elements (hyphae and spores), and a quantitative assessment of detected pathogens. This integrated system aims to translate the high analytical performance of the model into a practical tool for support diagnostic decision-making.

**Figure 8:**
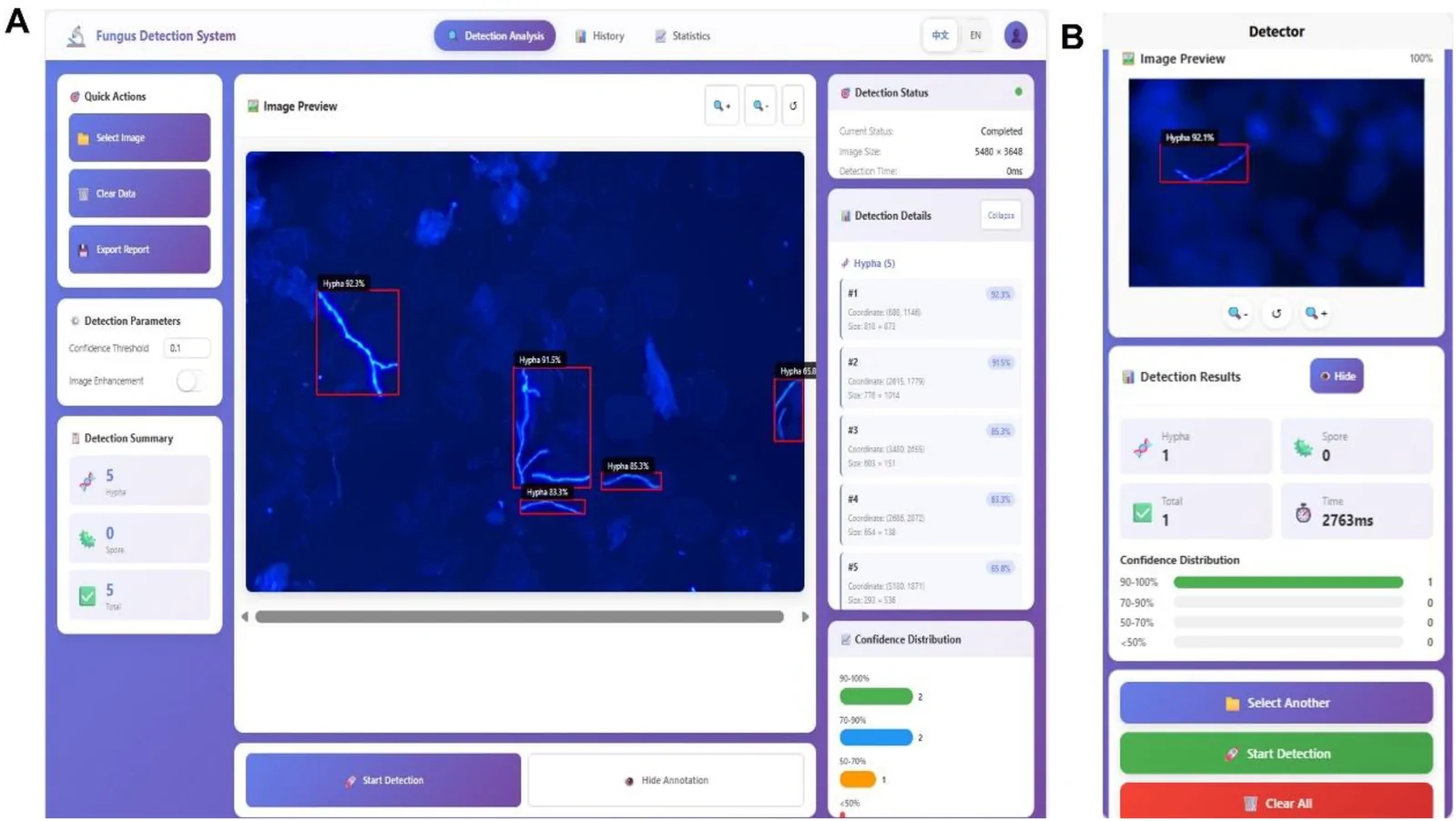
An online auxiliary diagnostic platform: **(A)** web-based interface on a desktop computer, **(B)** mobile interface on a smartphone.

## 4 Discussion

In this study, we systematically benchmarked six deep learning architectures for the microscopic detection of fungal structures and identified YOLOv11 as the optimal model through multi-level evaluation.

Accordingly, we established a multi-tier framework comprising three specialized datasets derived from 1, 870 clinical specimens. This extensive data foundation allowed us to evaluate AI models from technical, image-level and patient-level perspectives. Within this comprehensive cohort, tinea cruris (n=453) and tinea corporis (n=411) were the most prevalent subtypes, a distribution pattern that aligns with prior epidemiological findings from Southern China (Chen et al., 2018).

In our multi-model comparison, YOLOv11 exhibited superior performance in microscopic fungal detection relative to other evaluated models. These findings are consistent with existing literature. For instance, Zhao et al. reported that YOLOv11 effectively detects small biological targets in agricultural imaging, outperforming models such as DETR and DINO (Zhao et al., 2025). Similarly, Awad et al. observed that YOLOv11 achieved 98.2% detection accuracy for acute lymphoblastic leukemia, marginally surpassing YOLOv8 (98.0%)-a trend consistent with the performance gains observed in our study (Awad and Aly, 2024).

Our results also revealed a competitive performance between YOLOv11 and YOLOv8. For instance, YOLOv8 (97.38%) achieved a higher PPV for spore detection at 400× magnification compared with YOLOv11 (97.13%), suggesting that newer architectures may not universally outperform predecessors across all specific metrics (Gašparović et al., 2023; Geetha and Hussain, 2024; Ali et al., 2025; Jiang and Zhong, 2025). Released in 2023, YOLOv8 remains a robust baseline due to its anchor-free detection head and Cross Stage Partial Bottleneck with 2 convolutions (C2f) modules, which collectively optimize gradient flow and feature representation (Jocher et al., 2023). This architectural stability likely accounts for its strong performance on specific high-contrast targets. However, YOLOv8 has inherent limitations in capturing global contextual dependencies. Owing to its predominantly convolutional structure, it focuses on local features, which may compromise performance when fungal elements are obscured by complex background noise or tissue artifacts.

By comparison, the superior overall performance of YOLOv11 may be related to architectural refinements such as the Cross Stage Partial Bottleneck with 3 convolutions version 2 (C3k2) and C2PSA (Cross-Stage Partial with Self-Attention) modules (Ultralytics, n.d.). The clinical utility of YOLOv11 in medical image analysis is further supported by a recent study on vaginal discharge specimens, in which it successfully segmented fungal morphologies from mobile device-acquired images (Pongpom et al., 2026). Furthermore, the strong classification performance of models such as ResNet18 in related tasks (F1 score=0.986) suggests the potential of a hybrid workflow: rapid preliminary screening using a classification model followed by targeted detection with YOLOv11 (Liu et al., 2024). Regarding other algorithmic frameworks, this study further highlights their inherent limitations in clinical applications. Although DETR demonstrated acceptable performance for spores at 400× magnification, it was inferior to YOLO-series models in detecting small or irregularly shaped objects. This limitation likely stems from DETR’s reliance on low-resolution feature maps, which inherently reduce its sensitivity to small targets (Zhu et al., 2020).

For Faster R-CNN, we observed a characteristic profile of high TPR coupled with low PPV. This “overly sensitive” screening behavior is driven by the Region Proposal Network (RPN), which efficiently generates high-quality candidate regions and substantially improves recall (Ren et al., 2017). While beneficial for initial screening, the low PPV introduces a meaningful risk of false positives in clinical diagnosis. In contrast, YOLOv11 effectively mitigates these drawbacks, achieving a superior balance between precision and recall that is essential for definitive clinical diagnostics. The clinical acceptability of such balanced AI outputs is supported by expert evaluations of segmentation performance, which received high ratings (average 4.25/5) in a comparable study (Liu et al., 2024).

The suboptimal TPR of SSD-VGG16 observed in our study is consistent with previous reports, emphasizing inherent weaknesses of the SSD architecture in small-object detection. For instance, Zhou et al. demonstrated that SSD-based networks yielded markedly low sensitivity of 10%–40% when identifying targets smaller than 3 mm (Zhou et al., 2020).

Notably, all models detected spores significantly better than hyphae. This finding is consistent with results from Ren et al. and reflects a well-recognized challenge in fungal image analysis: the slender, variable morphology of hyphae makes them easily confused with background artifacts (Ren et al., 2025). However, this contrasts with observations by Gao et al., who reported higher specificity for hyphae (98%) than for spores (96%) (Gao et al., 2021). As illustrated in **Figure 9**, we analyzed the sources of validation errors in hyphae detection. The main confounding factors included:

**Figure 9:**
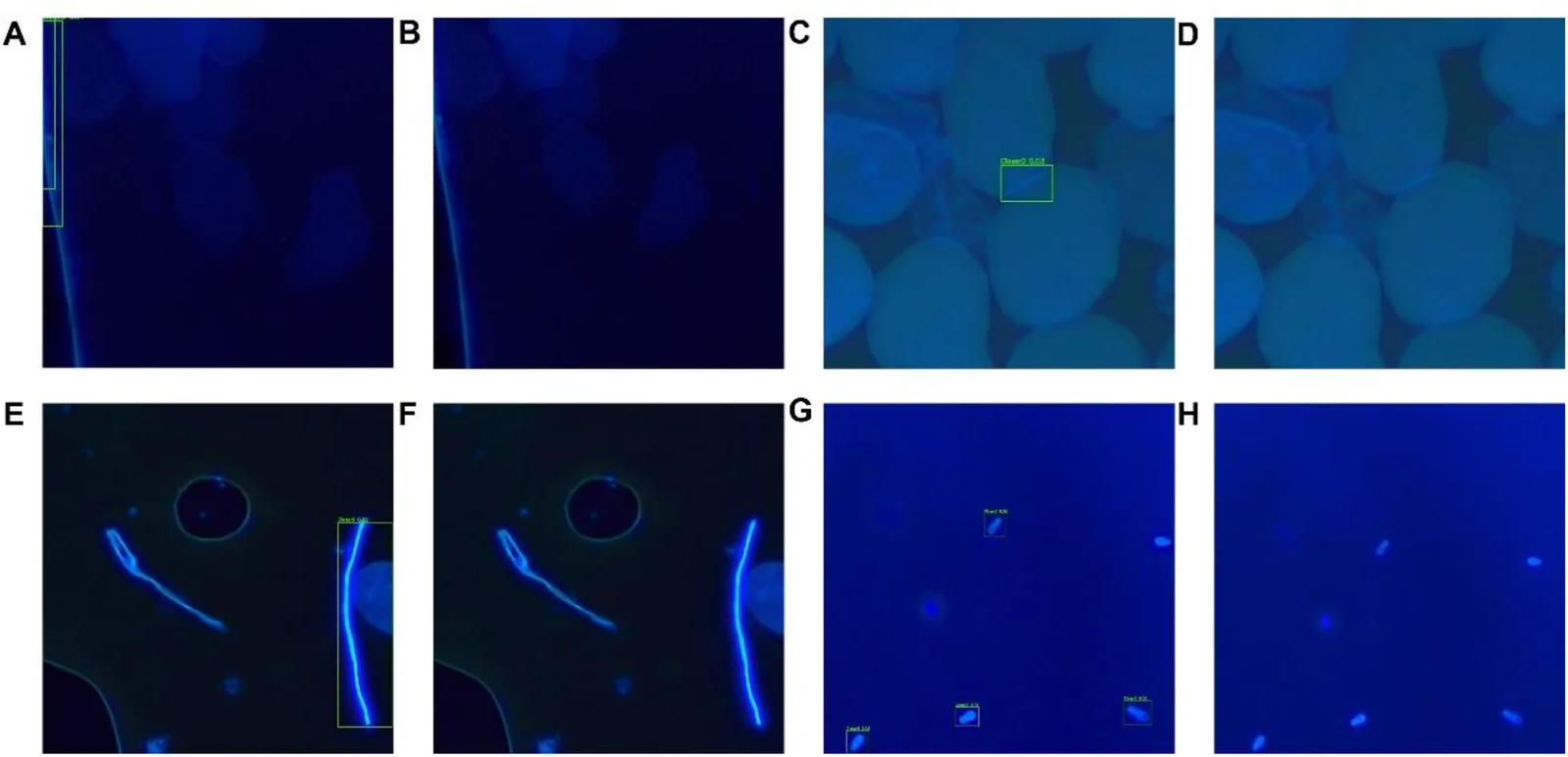
Typical examples of false detections. Typical examples of misidentification involving: **(A, B)** air bubbles, **(C, D)** human tissue artifacts, **(E, F)** fibrous materials, **(G, H)** *Malassezia* spores.

1. Fibrous artifacts and other negative background components, which accounted for most misidentifications;
2. *Malassezia* spores, associated with pityriasis versicolor, which resemble short hyphal segments;
3. Linear artifacts at the edges of air bubbles, which were misclassified as hyphal walls;
4. Interference from human tissue debris.

Furthermore, our results indicate that 400× magnification outperforms 200× across most metrics, supporting the notion that higher magnification facilitates more precise morphological characterization (Han et al., 2025). This may be because high magnification better captures fine structural details, whereas low magnification may obscure distinction between normal and pathological features. This principle is crucial for AI models, as demonstrated in high-resolution whole-slide image analysis systems for fungal elements (Assaf et al., 2025). By contrast, Ashtaiwi et al. reported that 200× magnification yielded better deep learning performance than 400× in breast cancer imaging (Ashtaiwi, 2022), suggesting a potential trade-off: while high magnification improves detail resolution, it may hinder feature detection when a wider field of view is required.

A key finding of this study is that a lower IoU threshold of 0.25 yields the optimal F1 score for fungal detection, in agreement with results reported by Koo et al (Koo et al., 2021). A lower IoU allows for greater tolerance in bounding box regression, which is particularly important for irregular, poorly defined targets such as hyphae. We also validated the Cmax strategy as the most effective image-level classification approach. Consistent with Koo et al (Koo et al., 2021), this aligned with the clinical “barrel effect” logic: the detection of even a single highly characteristic fungal structure is sufficient to confirm infection—a principle also applied in AI-enabled diagnostic platforms for other specimen types (Li et al., 2025).

In subgroup analysis, the model achieved the highest diagnostic efficacy for tinea capitis (F1 = 98.53%), likely due to the abundant spores typically present in tinea capitis samples, which display more distinct and recognizable features for the model compared to hyphae (Elewski, 2000). Conversely, tinea manuum exhibited the lowest efficacy, with an F1 score of 80.25%, likely attributable to the limited sample size (100 images), which restricted the model’s ability to learn discriminatory features (Khandaker et al., 2025). With F1 scores exceeding 90% for most tinea subtypes, YOLOv11 demonstrates robust diagnostic performance across diverse infection types. These results align with recent automated fluorescence microscopy findings (He et al., 2025), and confirm the superior performance of AI-driven systems in detecting superficial fungal infections, consistent with high performance of deep learning models in classifying microscopic fungal images (Zieliński et al., 2020).

This study has several limitations. First, the current platform requires manual image capture and does not automate the localization of fungal elements within full specimens. Future iterations should integrate whole-slide imaging (WSI), automated slide scanning, or microscope-integrated image acquisition systems to enable autonomous analysis of complete slides without manual representative image selection, as demonstrated by recent advances in high-speed microscopy (Liu et al., 2024) and WSI-based fungal detection (Assaf et al., 2025). Second, future multi-center external validation, as employed in other AI-based diagnostic studies (Li et al., 2025), is warranted to establish generalizability. Third, the ground truth was based on expert microscopic examination rather than fungal culture or polymerase chain reaction (PCR), meaning that inherent human interpretive errors may be propagated into model training. Incorporating culture or molecular methods as a reference standard in future work would strengthen the validity of the AI model, consistent with validation strategies used in modern rapid diagnostic platforms (Li et al., 2025). Fourth, this study did not include a direct comparison between the AI model and experienced human observers. While the patient-level results demonstrate high diagnostic accuracy, a prospective head-to-head comparison with clinicians of varying experience levels is needed to determine the added clinical value of the platform. Finally, the study is based on retrospective data from a single center, and prospective clinical validation is essential before clinical implementation. Despite these limitations, the performance of our model and its alignment with emerging AI applications in clinical microbiology underscore the transformative potential of deep learning in developing rapid, accurate and accessible tools for diagnosis of superficial fungal infections (Liu et al., 2024; Sroka-Oleksiak et al., 2025; Pongpom et al., 2026).

## 5 Conclusion

The results of this study showed that, through comparative evaluation of six deep learning architectures on a comprehensive single-center dataset, YOLOv11 was identified as the optimal model, demonstrating superior detection precision and computational efficiency. Targeted parameter optimizations-specifically an IoU threshold of 0.25 and the Cmax confidence strategy-significantly improved performance. The model exhibited robust detection across multiple tinea subtypes, with the highest accuracy achieved for tinea capitis. The development of an online auxiliary diagnostic platform demonstrates the feasibility of integrating deep-learning models into clinical workflows as a standardized tool to support real-time diagnostic decision-making. However, prospective multi-center validation, external validation, and direct comparison with clinician performance are essential before clinical implementation. This work represents a step toward combining advanced computer vision with clinical mycology to improve diagnostic accessibility and efficiency.

## Data Availability

The raw data supporting the conclusions of this article will be made available by the authors, without undue reservation.
